# Long Segment Intestinal Invagination in an Adult Case

**DOI:** 10.5005/jp-journals-10018-1225

**Published:** 2017-05-05

**Authors:** Elif Karadeli, Alper Parlakgumus, Sermin Tok, Gurcan Erbay

**Affiliations:** 1 Department of Radiology, Baskent University, Adana, Turkey; 2 Department of General Surgery, Baskent University, Adana, Turkey; 3 Department of Radiology, Faculty of Medicine, Mersin University, Mersin, Turkey

**Keywords:** Adult, Invagination, Long.

## Abstract

**How to cite this article:** Karadeli E, Parlakgumus A, Tok S, Erbay G. Long Segment Intestinal Invagination in an Adult Case. Euroasian J Hepato-Gastroenterol 2017;7(1):99-100.

Dear Editor,

In pediatric ages, invagination is one of the major causes of acute abdominal disorders.^1^ This is the intertwining of two consequent segments of one gastrointestinal segment. It is rare among adults. It is frequently observed in the small intestines.^2^ Our case was a 21-year-old female, who was admitted to our hospital with complaints of abdominal pain, nausea, and vomiting for 5 days and nondefecating problem for 1 day. The history included appendectomy 3 years previously and severe abdominal pain experienced from time to time for 3 years. The physical examination revealed rebound and diffuse sensitivity in the abdomen, being prominent in the upper right and lower right quadrants of the abdomen. The laboratory findings were as follows: C-reactive protein 17.40 mg/L, sedimentation 32 mm/hour, fibrinogen: 4.85 gm/L. Intravenous contrasted computed tomography (CT) imaging was performed due to the abdominal pain. In the CT imaging, long segment invaginated intestinal segment was observed, starting in the ascending colon level in the lower right quadrant, fitting the transverse colon level, and partially extending to the descending colon level (Fig. 1). In the surgical exploration, the cecum and transverse colon were observed invaginated, and right hemicolectomy and peripheral-side ileotransverse colostomy were performed. No benign or malignant finding that may cause invagination was observed in the pathology material. As a conclusion, invagination is rare among adults, and CT is the golden standard for the diagnosis.

**Fig. 1:**
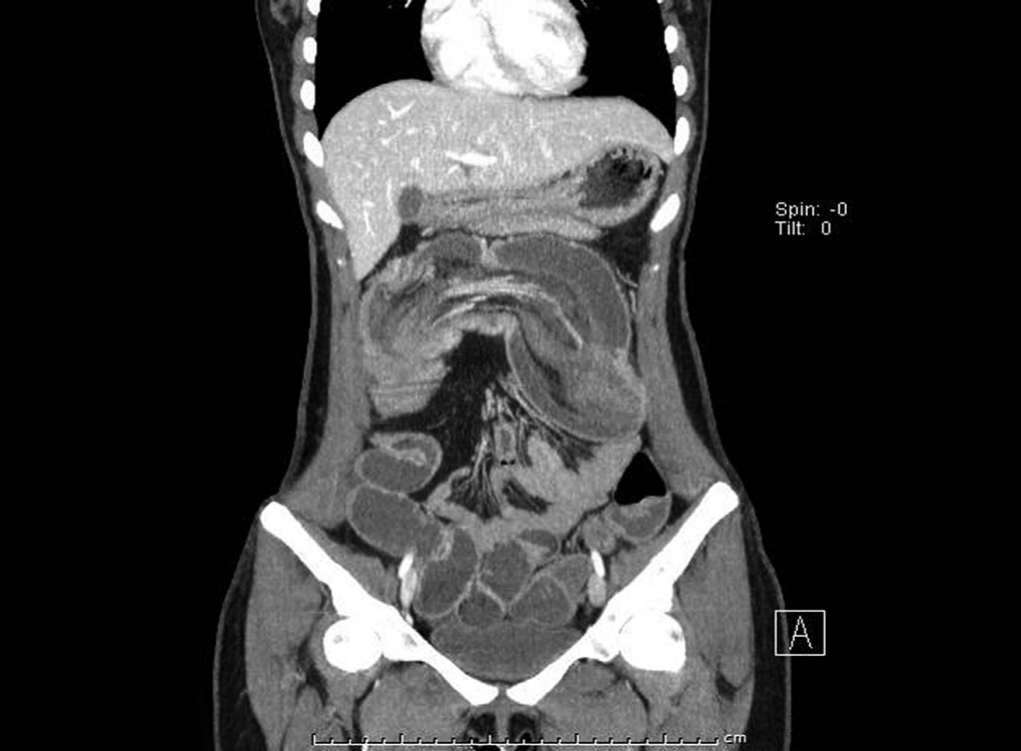
Coronal CT image; long segment invaginated intestinal segment was observed starting in the ascending colon level in the lower right quadrant, fitting the transverse colon level and partially extending to the descending colon level

In 70 to 90% of the adults, the pathology may be defined for invagination. The etiology includes benign reasons, such as hamartoma, lipoma, leiomyoma, inflammatory adenoma, or Meckel’s diverticulum. Malignancy is common among colon invaginations.^3^

The clinical presentation in adults is generally chronic or nonspecific. Invagination frequently presents with acute abdominal pain, nausea, vomiting, and high white blood cell count in adults.^1^ The most common complaint is abdominal pain. Nausea and vomiting are the second common complaint. Palpable mass may be observed in the abdomen.^4^ False kidney appearance in the sonographic longitudinal plan and target appearance in the transverse plan are diagnostic. The golden standard is CT imaging in the diagnosis, and it may also define the etiology. The treatment of the invagination is surgical in adult cases.^2^
